# Bisphosphonates for the Prevention and Treatment of Osteoporosis in Patients with Rheumatic Diseases: A Systematic Review and Meta-Analysis

**DOI:** 10.1371/journal.pone.0080890

**Published:** 2013-12-06

**Authors:** Zhiyun Feng, Shumei Zeng, Yue Wang, Zhiyun Zheng, Zhong Chen

**Affiliations:** 1 Spine lab, Department of Orthopedic Surgery, The First Affiliated Hospital, College of Medicine, Zhejiang University, Hangzhou, Zhejiang, China; 2 Department of Gynaecology and Obstetrics, Xiaolan People’s Hospital Affiliated to Southern Medical University, Zhongshan, Guangdong, China; 3 Key Laboratory of Organ Transplantation, Hangzhou, Zhejiang, China; National Taiwan University, Taiwan

## Abstract

**Background:**

While bisphosphonates (BPs) are commonly used in clinical treatment for osteoporosis, their roles on osteoporosis treatment for rheumatic patients remain unclear. We performed a meta-analysis to evaluate the efficacy of BPs on fractures prevention and bone mass preserving in rheumatic patients.

**Methodology/Principal Findings:**

We searched PubMed, EmBase, and the Cochrane Central Register of Controlled Trials for relevant literatures with a time limit of Jan. 6, 2012. All randomized clinical trials of BPs for adult rheumatic patients with a follow-up of 6 months or more were included. We calculated relative risks (RRs) for fractures and weighted mean difference (WMD) for percent change of bone mineral density (BMD). Twenty trials were included for analysis. The RR in rheumatic patients treated with BPs was 0.61 (95%CI [0.44, 0.83], P = 0.002) for vertebral fractures, and 0.49 (95%CI [0.23, 1.02], P = 0.06) for non-vertebral fractures. The WMD of BMD change in the lumbar spine was 3.72% (95%CI [2.72, 4.72], P<0.001) at 6 months, 3.67% (95%CI [2.84, 4.50], P<0.001) at 12 months, 3.64% (95%CI [2.59, 4.69], P<0.001) at 24 months, and 5.87% (95%CI [4.59, 7.15], P<0.001) at 36 months in patients using BPs, as compared with those treated with calcium, vitamin D or calcitonin. In subgroup analyses, rheumatic patients using BPs for osteoporosis prevention had greater WMD than those using BPs for treating osteoporosis at 6 months (4.53% vs. 2.73%, P = 0.05) and 12 months (4.93% vs. 2.91%, P = 0.01).

**Conclusions/Significance:**

In both short-term and middle-term, BPs can preserve bone mass and reduce the incidence of vertebral fractures in rheumatic patients, mainly for those who have GC consumption. The efficacy of BPs is better when using BPs to prevent rather than to treat osteoporosis in rheumatic patients.

## Introduction

Rheumatic diseases are inflammatory conditions which may cause significant swelling and pain in joints and muscles, resulting in a decreased life quality or even disability. Most of rheumatic diseases could be attribute to the over-activated immune responses. Glucocorticoid (GC) is one of the fundamental medicines to suppress such hyperactive inflammation for most rheumatic diseases, such as rheumatoid arthritis (RA) and systemic lupus erythematosus (SLE). Although GC may substantially control rheumatic symptoms and slow down disease progression, it is a major factor responsible for prominent osteoporosis in rheumatic patients. GC can reduce bone formation and inhibit intestinal calcium absorption, leading to secondary hyper-parathyroidism and increased osteoclastic bone activities, which ultimately result in secondary osteoporosis [1].

As rheumatic-related osteoporosis and fractures may substantially deteriorate the original rheumatic illness and increase the cost of health care [2], the prevention and treatment of osteoporosis are important clinical issues in managing rheumatic diseases. To date, however, the duration and dosage of GC that may trigger bone loss are still controversial. While low dose GC consumption (prednisolone, <7.5 mg/day) was reportedly inadequate to induce significant osteoporosis clinically [3], the bone loss rates could as high as 13.9% per year in patients treated with high dosage of GC (prednisolone, >7.5 mg/day) [4], [5]. Some other studies, however, reported that patients taking low dosage of GC had an increased risk of both vertebral and non-vertebral fractures [6], [7]. Furthermore, the fracture risk was more related to daily dose than cumulative dose of GC [6], [7].

It should be acknowledged that GC consumption is not the only contributor of bone loss in rheumatic patients. A number of other factors, such as severity of rheumatic diseases, immobility, disease-modifying drugs, and inflammatory factors are also involved in the pathogenesis of bone loss [8]–[14]. For example, longitudinal studies observed that bone loss was more common in patients with SLE or RA, suggesting that SLE or RA may be an independent factor for secondary osteoporosis [4], [14].

Bisphosphonates (BPs), a family of anti-osteoporosis drugs with strong inhibitory effects on osteoclastic bone osteoporotic, acts as a potential candidate for modifying bone loss in rheumatic patients [15], [16]. In 2000, Homik and colleagues [17] performed a meta-analysis of 13 randomized controlled trials (RCTs) of BPs in the prevention and treatment of glucocorticoid-induced osteoporosis. By that time, however, the second or third generations BPs, such as alendronate and ibandronate, have not been widely used in clinical practice. Moreover, among the included trials most BPs used was etidronate and the longest follow-up time was 12 months. In addition, the included patients suffered from a variety of diseases requiring GC treatment rather than only rheumatic diseases, such as asthma, chronic obstructive pulmonary disease, organ transplantation, and inflammatory bowel diseases. In studying the association between GC usage and bone mineral density (BMD) change, one had better take the primary disease into consideration as it may be a strong confounder [18].

On the other hand, whether BPs can reduce the fracture risk in rheumatic patients remains unclear. While Frediani et al [19] observed that clodronate can effectively reduce the incidence of vertebral fractures in patients with RA and psoriatic arthritis, Eastell et al [20] and Lems et al [21] reported that risedronate and alendronate had no effect on the prevention of vertebral fractures in rheumatic patients. In another study, rheumatic patients taking alendronate were less likely to experience new vertebral deformities than those taking alfacalcidol [22].

It is clinically important to determine the efficacy of BPs on the prevention and treatment of osteoporosis in patients with rheumatic diseases. While the second and the third generation of BPs are common medicines now, an updated review on BPs in the prevention and treatment of osteoporosis for rheumatic patients is absent. Thus, we performed a comprehensive meta-analysis with the inclusion of RCTs of middle-term follow-up to summarize current evidence and guide related clinical practice.

## Methods

### Literature Search

Two authors (ZYF and SMZ) independently searched PubMed, EMBASE, and the Cochrane Central Register of Controlled Trial, with the article type restricted to clinical trial and a time limit of Jan. 6, 2013. No other limits were used. The terms used in search were: bisphosphonates, BPs, etidronate, alendronate, zoledronate, neridronate, olpadronate, clodronate, pamidronate, incadronate, tiludronate, ibandronate, neridronate. These terms were used in combination with each of the following medical headings: rheumatic diseases, rheumatoid arthritis, RA, psoriatic arthritis, PsA, systemic lupus erythematosus, SLE, ankylosing spondylitis, AS, polymyositis, dermatomyositis, systemic sclerosis, vasculitis syndrome, still’s disease, polymyalgia rheumatic, PMR, systemic sclerosis, Sjögren’s syndrome, and Behcet’s disease. In addition, studies of BPs in the treatment and prevention of glucocorticoid-induced osteoporosis were also reviewed. The identified RCTs were included in the present study only if all or most of participants suffered from rheumatic diseases. Conference abstracts of American College of Rheumatology, International Osteoporosis Foundation, and American Society for Bone and Mineral Research were further searched to identify additional data, if any. Additionally, Google Scholar Search was used to identify studies that were missed in searching academic databases.

### Selection Criteria

(1) Types of study: Only RCTs were selected for further assessment because observational studies are more likely to have confounding bias. Trials focusing on the comparison of different BPs, or between BPs and denosumab or teriparatide were not included. (2) Participants: Only ambulatory rheumatic patients older than 18 years were included, regardless of gender and menopausal status. (3) Intervention: The intervention was the use of any generation of BPs, alone or together with calcium and/or vitamin D, irrespective of administered approach. The intervention in control group was placebo, alone or together with calcium, vitamin D, and calcitonin. (4) Outcomes: Incidence of vertebral and non-vertebral fractures was collected as the primary outcome. Percent change of BMD measured by dual-energy X-ray absorptiometry (DXA) at lumbar spine, total hip and femoral neck at 6, 12, 24, and 36 months were the secondary outcome.

### Quality Assessment and Data Extraction, Conversion, and Analysis

The identified studies were reviewed by 2 investigators (ZYF and ZC) independently with a Jadad score table [23]. This table contents items regarding randomization (2 points), blinding (2 points), and description of withdrawals (1 point). We also contacted the first or the corresponding author for further information. If evaluation scores were different between two raters, the study was further discussed to reach an agreement.

Two authors independently extracted all the related data which were further checked by the first author (ZYF) for the accuracy. The extracted data included demography (number of participants, average age, gender, and original diseases), intervention details (duration, protocol, and follow-up time), percent change of BMD at lumbar spine, total hip, and femoral neck, fracture incidence, number of withdrawals due to side-effects, number of patients experiencing gastrointestinal symptoms. If needed, further contact with the corresponding author was tried for more details.

Intention-to-treat data were used whenever possible. If absent, per-protocol data or available analyzing data were used. When standard deviations (SDs) were not presented in the paper, standard error of the means (SEMs) and 95% confidence intervals (95%CIs) were transferred into SDs. If P values and means were presented, we converted P value to Z score to calculate SEM using “Z = mean difference/standard error”. If necessary, we extrapolated means, SEMs or SDs from the available graphs and tables. If a trail has two intervention protocols (e.g.: daily and cyclical), we only used one intervention group. If a trial has two control groups, we combined the data of both, as described in a previous reference [24].

### Statistical Analysis

Pooled analysis for fracture incidence was conducted using Mantel-Haenszel relative risk (RR). Results were presented as RRs and 95% CIs. As GC may have different influences on cortical and trabecular bones, percent change of BMD at hip and lumbar spine at 6, 12, 24, and 36 months were analyzed separately. Weighted mean differences (WMDs) between BPs groups and control groups were calculated as overall treatment effects for the combined trials and were presented together with their 95% CIs.

Heterogeneity among the outcomes of combined trials were tested using both chi square statistic and heterogeneity I^2^ statistic on N−1 degrees of freedom with substantial heterogeneity defined as greater than 50%. Effect size estimates were analyzed using fixed effects models if the data were homogeneous. Otherwise random effects models were used. Subsequently, subgroups analysis, based on manner of BPs therapy (prevention or treatment), generation of BPs (the first generation (G1) or the second and the third generation (G2-3)), usage of BPs (continuous or intermittent), calcium supplement, type of diseases (RA only or not), and mean age of patients (younger than 50 or older than 50), were performed to identify factors influencing efficacy of BPs. Also, sensitive analyses were performed to evaluate the robustness of the results, the analyses examined the effects of methodological quality (Jadad score and blinding). Funnel plots with Egger’s test and Begg’s test were used to evaluate the publication bias. If plots were asymmetrical, then trim and fill analyses were performed to evaluate the stability in overall effects. All the reported P values were two-side and a P value less than 0.05 was considered as statistically significant. Statistical analyses were performed in Review Manager 5.2 (RevMan, Version 5.2, The Cochrane Collaboration, Copenhagen) and STATA (version 12.0, Stata Corp, College Station, Texas).

## Results

### Literature Search

Initially, there were 467 relevant trials identified. After a preliminary review, 410 papers were excluded because of duplication or irrelevancy. The remaining 57 trials were closely reviewed. Among them, 18 trials were not RCTs, 9 trials were not studying adults, and another 4 trials were duplicated, and therefore were further excluded, leaving 26 eligible trials. Of these 26 trials, data of BMD in 3 studies were not normally distributed [25]–[27]; BMD was measured by microdensitometry method and p value or SEM was not reported in 2 trials [28], [29]; absolute values of BMD rather than percent changes of BMD were presented in another 2 trials [30], [31]. These papers were excluded as calculation of SDs and the percent change of BMD were not feasible. In addition, we obtained 2 abstracts fulfilled with inclusion criteria in abstract books and conference proceedings [32], [33], but one [32] did not mention numbers of patients in each group and the authors did not respond to our email contacts. Therefore, only one was included.

**Figure 1:**
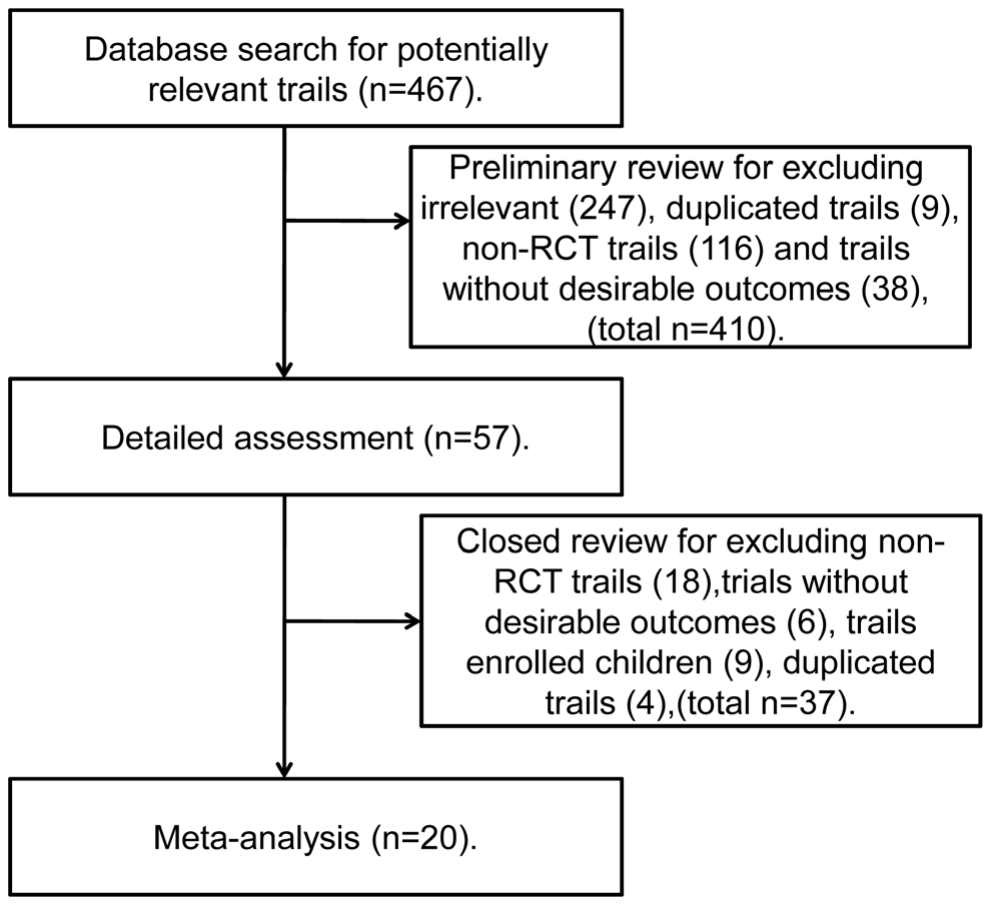
Diagram of literature search and selection.

As a result, there were 20 trials included in the current meta-analysis (Figure 1). Among the 20 trials, three also included some non-rheumatic patients (4 patients in the Roux’s study (4/117), 2 patients in the Boutson’s study (2/27), 8 patients in Adachi’s study (8/141)) [34]–[36]. These 3 trials were included as the vast majority of their participants were patients with rheumatic diseases. Funnel plots suggested that there was no statistically significant publication bias among trials reporting new fractures incidence (Egger’ test P = 0.50, Begg’s test P = 0.28) (Figure S1). The publication bias among studies reporting percent change of BMD, however, was significant (Egger’ test P = 0.01, Begg’s test P = 0.03). The trim and fill analysis found 6 potentially unpublished trials but the overall effects were not substantially influenced.

### Study Characteristics

The characteristic of the included studies was summarized in **Table S1**. The data consisted of 1422 patients with rheumatic diseases, with 713 patients randomized to BPs group and the other 709 to control group. The sample size ranged from 12 to 201 patients. Included patients were restricted to RA in 5 trials [20], [21], [37]–[39]. The average age of patients was more than 50 years in 14 trials [19]–[22], [34], [36]–[44], and younger than 50 years in 6 trials [33], [45]–[49]. Eight trials were prevention trials, defined as starting BPs treatment in the first three months of GC therapy [22], [33], [34], [36], [41], [42], [44], [45]. Eleven trials were classified as treatment trials because BPs was given for long-term GC user. Of these 11 treatment trials, the mean dosage of GC consumption was greater than 7.5 mg/day (prednisone equivalent) in 6 trials [19], [38], [46]–[49], less than 7.5 mg/day in 5 trials [20], [21], [39], [40], [43]. No GC usage in 1 trial [37]. calcium and vitamin D was given to patients in 10 trials [19], [21], [22], [38]–[40], [43], [45]–[47], only calcium in 5 trials [34], [36], [41], [42], [49], only placebo in 5 trial [20], [33], [37], [44], [48].

### Quality Assessment

According to Jadad score table, 2 trials had a score of 5 points, 6 trials had a score of 4, 5 trials had a score of 3, and 5 trials had a score of 2, 2 trials had a score of 1 (Table S1). All the included trials were randomized and 9 were double blinded (Table S1). We contacted authors for detailed study information, including randomization, blinding, and description of withdrawals for Jadad score evaluation in 9 trials [19]–[21], [36], [38], [39], [43], [48], [49]. Only authors of three papers responded with favorable information [20], [43], [49]. Intention-to-treat analyses were used in most of trials. The weighted kappa for the agreement of the Jadad score between two investigators was 0.80 [0.72, 0.97].

### Vertebral Fractures

**Figure 2:**
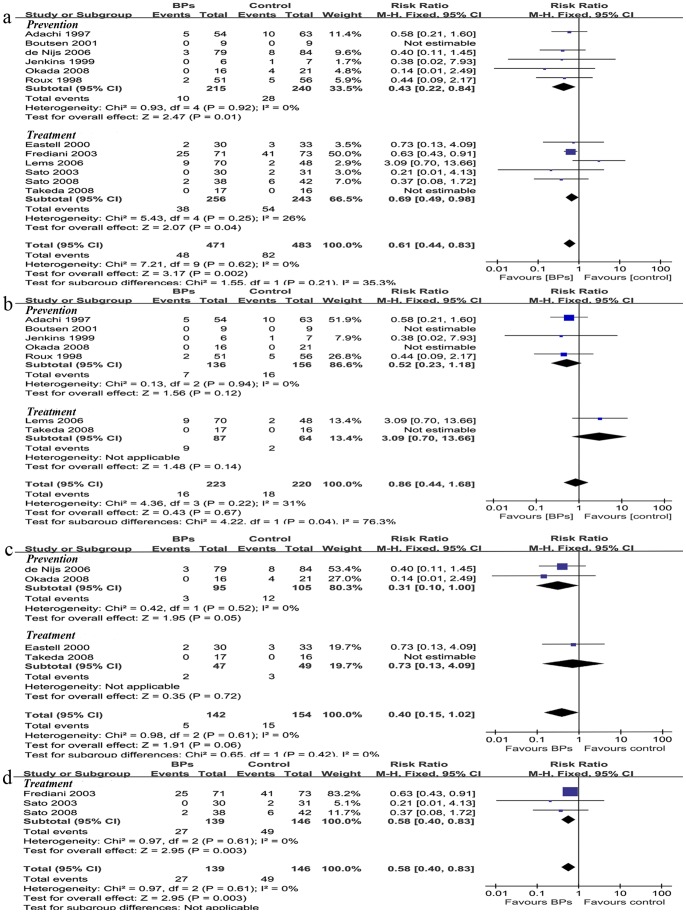
Biophosphonates for vertebral fracture of rheumatic patients. Pooled estimate for the relative risk of vertebral fractures (a) and relative risk of vertebral fractures at 12 months (b), 24 months (c), and 36 months (d) showed that BPs reduce the risk of vertebral fractures in rheumatic patients. BPs, bisphosphonates; Prevention, trials starting BPs treatment in the first three months of GC therapy, Treatment, trials giving BPs for long-term GC user; 95% CIs, confidence intervals; Boxes, estimated risk ratios; bars, 95% CIs, diamonds, pooled RRs; width of diamonds, pooled CIs.

Ten trials (n** = **903) reported incidence of vertebral fractures at 12, 18, 24, and 36 months. Two prevention trials reported vertebral fractures at 18 months, and we combined that with data of 24 months. All the included rheumatic patients of the ten trials were treated by GC. We combined symptomatic and asymptomatic vertebral fractures for analysis. The estimate RR for vertebral fractures was 0.61 (95%CI [0.44, 0.83], P = 0.002) (Figure 2a, Table 1). When the prevention and treatment subgroup were analyzed separately, the RR was 0.43 (95%CI [0.22, 0.84], P = 0.01) and 0.69 (95%CI [0.49, 0.98], P = 0.04), respectively (Figure 2a). The efficacy of BPs at different time-points was reported in **Table 1**. A statistically significant RR was observed only in 18-month follow-up in prevention group (P = 0.05) (Figure 2c), and 36-month and longer follow-up in treatment group (P = 0.003) (Figure 2d).

**Table 1:** Summary of effects of BPs on patients with rheumatic disease at different time-points.

	6 months			12 months			24 months			36 months		
	N	WMD [95%CI]	P	N	WMD [95%CI]	P	N	WMD [95%CI]	P	N	WMD [95%CI]	P
Lumbar spine	11	3.72 [2.72,4.72]	0.001	19	3.67 [2.84,4.50]	0.001	6	3.64 [2.59,4.69]	0.001	4	5.87 [4.59,7.15]	0.001
Total hip	4	0.81 [0.22,1.39]	0.007	7	2.23 [1.29,3.17]	0.001	2	5.90 [5.61,6.19]	0.001	1	7.48 [7.14,7.82]	0.001
Femoral neck	6	1.36 [0.74,1.99]	0.01	10	2.46 [1.75,3.18]	0.001	4	3.58 [0.68,6.47]	0.02	2	4.15 [−0.38,8.67]	0.07
VF	N.A.			7	0.98 [0.52,1.86]^a^	0.95	4	0.4 [0.15,1.02]^a^	0.06	3	0.58[0.40, 0.83]^a^	0.003
					Analysis for trails at all time-points, n = 954, N = 12, RR = 0.61 [0.44, 0.83], P = 0.002.							
NVF	N.A.			5	0.49[0.23, 1.02]^a^	0.06	N.A.			N.A.		
				Analysis for trails at all time-points, n = 734, N = 5, RR = 0.49 [0.23, 1.02], P = 0.06.								

VF, Vertebral fracture; NVF, Non-vertebral fracture; N.A., not available; WMD: weighted mean difference; 95%CI: 95% confidential interval; RR: relative risk;

^a^ expressed as RR [95%CI].

### Non-vertebral Fractures

**Figure 3:**
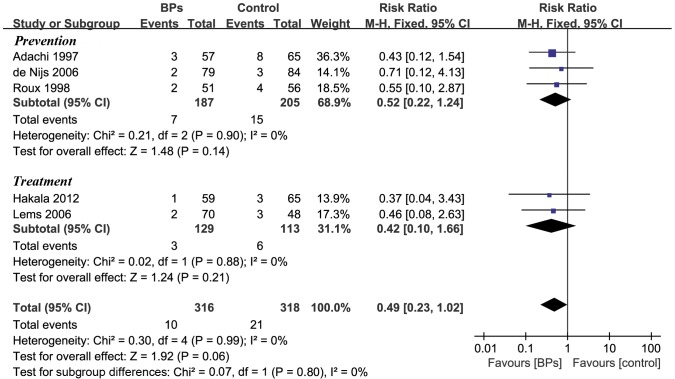
Biophosphonates for non-vertebral fracture of rheumatic patients. Pooled estimate for the relative risk of non-vertebral fractures showed that rheumatic patients using BPs tended to have a lower incidence of non-vertebral fracture than those not taking BPs, though this tendency did not reach statistical significance in the current study. BPs, bisphosphonates; Prevention, trials starting BPs treatment in the first three months of GC therapy, Treatment, trials giving BPs for long-term GC user; 95% CIs, confidence intervals; Boxes, estimated risk ratios; bars, 95% CIs, diamonds, pooled RRs; width of diamonds, pooled CIs.

Five trials (n = 634) reported the incidence of non-vertebral fractures. All the included rheumatic patients of the five trials were treated by GC. Among them, three reported 12 months data and the other two 18 months data. The combined data showed the RR for non-vertebral fractures in BPs group was 0.49 (95%CI [0.23, 1.02], P = 0.06) (Figure 3), as relative to control group. The RR and 95% CI for the prevention and treatment subgroup were reported in **Figure 3**.

### Percent Change of BMDs in the Lumbar Spine, Total Hip and Femur Neck

All the trials reported BMD data; one of them included patients without GC treatment. In rheumatic patients taking high dosage of GC, while significant bone loss at these sites was observed when they were treated with calcium and/or vitamin D, those treated with BPs had a slight bone loss, or even had a bone accrual.

**Figure 4:**
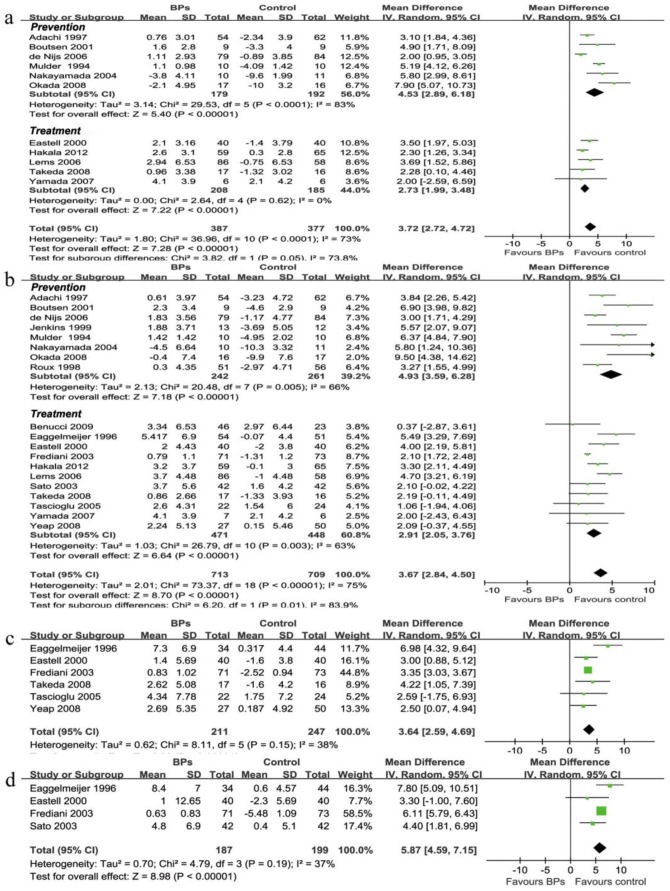
Biophosphonates for percent change of bone mineral density of rheumatic patients. Weighted mean difference of percent change of lumbar spine BMD at 6 months (a), 12 months (b), 24 months (c), and 36 months (d) showed that BPs significantly prevent bone loss at lumbar spine, this efficacy increases over time during the middle-term follow-up. BPs, bisphosphonates; Prevention, trials starting BPs treatment in the first three months of GC therapy, Treatment, trials giving BPs for long-term GC user; 95% CIs, confidence intervals; WMD, weighted mean difference; Boxes, weighted mean difference; bars, 95% CIs, diamonds, Pooled WMDs; width of diamonds, pooled CIs.

Combining available data at 6 months (11 trials, n = 764, WMD = 3.72%, 95%CI [2.72, 4.72], P<0.001); 12 months (19 trials, n = 1317, WMD = 3.67%, 95%CI [2.84, 4.50], P<0.001); 24 months (6 trials, n = 431, WMD = 3.64%, 95%CI [2.59, 4.69], P<0.001); and 36 months (4 trials, n = 386, WMD = 5.87%, 95%CI [4.59, 7.15], P<0.001) all showed significant preserve in lumbar spine BMD in favor of BPs (Figure 4, Table 1).

Combining available data at 6 months (4 trials, n = 449, WMD = 0.81%, 95%CI [0.22, 1.39], P<0.01); 12 months (7 trials, n = 716, WMD = 2.23%, 95%CI [1.29, 3.17], P<0.001); 24 months (2 trials, n = 221, WMD = 5.9%, 95%CI [5.61, 6.19], P<0.001); and 36 months (1 trials, n = 144, WMD = 7.48%, 95%CI [7.14, 7.82], P<0.001) all showed significant preserve in hip BMD in favor of BPs (Table 1).

Combining available data at 6 months (6 trials, n = 529, WMD = 1.36%, 95%CI [0.74, 1.99], P<0.01); 12 months (10 trials, n = 715, WMD = 2.46%, 95%CI [1.75, 3.18], P<0.01); 24 months (4 trials, n = 281, WMD = 3.58%, 95%CI [2.59, 4.69], P<0.01); and 36 months (2 trials, n = 158, WMD = 4.15 [−0.38, 8.67], P = 0.07), all but the 36 months follow-up showed significantly preserve in femur neck BMD in favor of BPs (Table 1).

### Heterogeneity

No statistically significant heterogeneity was observed among the 10 trials reporting vertebral fracture (χ^2^ = 7.21, df = 9, P = 0.62, I^2^ = 0%) and the 5 trials involving non-vertebral fracture (χ^2^ = 0.3, df = 4, P = 0.99, I^2^ = 0%). For the percent change of BMD, however, significant heterogeneity was noticed at some time-points.

For the percent change of lumbar spine BMD outcome, heterogeneity was observed at 6 months (χ^2^ = 36.96 df = 10, P<0.01, I^2^ = 73%) and 12 months (χ^2^ = 73.37 df = 18, P<0.01, I^2^ = 75%), but not at the other two time-points. They cannot be fully explained by factors mentioned in the prior text. When we classified data into subgroups by manner of BPs therapy (prevention or treatment), the heterogeneity decreased: for prevention trials, (χ^2^ = 2.64, df = 4, P = 0.62, I^2^ = 0%) at 6 months and (χ^2^ = 20.48, df = 7, P = 0.005, I^2^ = 66%) at 12 months); for treatment trials, (χ^2^ = 29.53, df = 5, P<0.01, I^2^ = 83%) at 6 months and (χ^2^ = 26.79, df = 10, P = 0.003, I^2^ = 63%) at 12 months). It should be noted that three prevention trials enrolled patients who started BPs therapy within the first three months of starting GC therapy [22], [34], [42], while the other five trials only enrolled patients who started BPs therapy at the first time of GC therapy [33], [36], [41], [44], [45]. When the data were divided according to inclusion criteria, the heterogeneity disappeared: trials enrolling patients starting GC consumption within early 3 months, (χ^2^ = 1.73, df = 1, P = 0.19, I^2^ = 42%) at 6 months and (χ^2^ = 0.66, df = 2, P = 0.72, I^2^ = 0%) at 12 months; trials enrolling patients starting GC consumption at first time, (χ^2^ = 3.27, df = 3, P = 0.35, I^2^ = 8%) at 6 months and (χ^2^ = 1.78, df = 4, P = 0.78, I^2^ = 0%) at 12 months.

Among the 11 treatment trials, 6 used GC in a high dosage; the other 5 used GC in a low dosage. When data were divided according to the mean dosage of GC, the heterogeneity in the treatment subgroup was also disappeared: low dosage of GC trials, (χ^2^ = 2.45, df = 3, p = 0.48, I^2^ = 0%) at 6 months and (χ^2^ = 0.46, df = 4, P = 0.98, I^2^ = 0%) at 12 months; high dosage of GC trials, (not applicable) at 6 months and (χ^2^ = 9.31, df = 5, P = 0.1, I^2^ = 46%) at 12 months.

For total hip, there was no consistent heterogeneity other than at 12 months time-point: (χ^2^ = 21.68, df = 6, P = 0.001, I^2^ = 72%). For femur neck, heterogeneity was observed at 24 months (χ^2^ = 10.6, df = 3, P = 0.01, I^2^ = 72%) and at 36 months (χ^2^ = 4.15, df = 1, P = 0.04, I^2^ = 76%). Similarly, the observed heterogeneity could be explained by the daily mean dosage of GC.

### Subgroup Analysis

Subgroup analyses were carried out for vertebral fractures and percent change of lumbar spine BMD at all time points. The prevention subgroup had a greater RR reduction for vertebral fractures: (RR = 0.43, 95%CI [0.22, 0.84] vs. RR = 0.69, 95%CI [0.49, 0.98], P = 0.21) (Figure 2a). Correspondingly, the efficacy of BPs on preserving lumbar spine BMD was greater in prevention subgroup than in treatment subgroup at both 6 and 12 months (P = 0.05 and P = 0.01, respectively) (Table 2). There were no prevention trials at 24 and 36 months time-points. Although BPs is less effective in decreasing the risk of vertebral fractures for RA patients than for patients with other rheumatic diseases, the efficacy on improving lumbar spine BMD was comparable (Table 2). No statistical difference was identified when subgroup analyses were performed based on the other factors (Table 2).

**Table 2:** Subgroup analysis for vertebral fractures and percent change of lumbar spine BMD at different time-points.

Factor	Vertebral fractures			6 months			12 months			24 months			36 months		
	N	WMD[95%CI]	P	N	WMD[95%CI]	P	N	WMD[95%CI]	P	N	WMD[95%CI]	P	N	WMD[95%CI]	P
M1	5	0.43[0.22,0.84]	N.S.	6	4.53[2.89,6.17]	0.05	8	4.93[3.59,6.28]	0.01		N.A.	N.A.		N.A.	N.A.
M2	5	0.69[0.44,0.98]		5	2.73[1.99,3.48]		11	2.91[2.05,3.76]		6	3.64[2.59,4.69]		4	5.87[4.59,7.15]	
U1	3	0.78[0.35,1.72]	N.S.	7	3.46[1.96,4.96]	N.S.	9	3.47[2.23,4.68]	N.S.	4	4.32[2.26,6.39]	N.S.	2	6.52[4.32,8.81]	N.S.
U2	7	0.57[0.41,0.80]		4	4.02[2.56,5.48]		10	3.63[2.51,4.75]		2	3.34[3.02,3.65]		2	6.09[5.77,6.40]	
G1	6	0.56[0.40,0.79]	N.S.	3	4.51[2.83,6.18]	N.S.	7	3.88[2.36,5.41]	N.S.	1	3.35[3.03,3.67]	N.S.	2	6.09[5.77,6.40]	N.S.
G2-3	4	0.77[0.38,1.59]		8	3.31[2.20.4.41]		12	3.57[2.59,4.55]		5	3.85[2.64,5.05]		2	6.52[4.32,8.81]	
Ca1	9	0.60[0.44,0.80]	N.S.	8	3.65[2.46,4.84]	N.S.	13	3.33[2.50,4.16]	N.S.	2	3.34[3.03,3.66]	N.S.	2	6.09[5.77,6.40]	N.S.
Ca2	1	0.73[0.13,4.09]		3	3.85[2.19,5.52]		6	4.61[2.84,6.83]		4	4.55[2.89,6.21]		2	6.52[4.32,8.81]	
RA	2	1.80[0.62,5,24]	0.03	3	3.46[2.25,4.66]	N.S.	5	3.62[2.66,4.58]	N.S.	3	4.30[2.75,5.85]	N.S.	2	6.52[4.32,8.81]	N.S.
RD	8	0.52[0.38,0.73]		8	3.88[2.61,5.16]		14	3.96[2.62,5.30]		3	3.34[3.03,3.66]		2	6.09[5.77,6.40]	
Age1	7	0.67[0.48,0.92]	N.S.	8	3.32[2.33,4.31]	N.S.	14	3.77[2.82,4.71]	N.S.	5	3.40[3.09,3.71]	N.S.	3	6.12[5.81,6.43]	N.S.
Age2	3	0.26[0.08,0.89]		3	5.23[1.48,8.62]		5	3.41[1.39,5.43]		1	2.51[0.07,4.94]		1	4.40[1.81,7.00]	
High	9	0.59[0.36,0.96]	N.S.	7	3.43[2.36,4.50]	N.S.	12	3.93[3.16,4.70]	N.S.	3	3.90[2.53,5.27]	N.S.	3	5.59[3.87,7.71]	N.S.
Low	1	0.63[0.43,0.91]		4	4.15[2.33,5.98]		7	2.87[1.05,4.69]		3	3.35[3.04,3.67]		1	6.11[5.79,6.43]	
B1	7	0.65[0.38,1.12]	N.S.	6	3.31[2.20,4.43]	N.S.	9	3.98[3.26,4.71]	N.S.	2	4.55[2.89,6.21]	N.S.	2	6.52[4.32,8.81]	N.S.
B2	3	0.58[0.40,0.83]		5	4.32[2.85,5.19]		10	3.08[1.68,4.89]		4	3.34[3.03,3.66]		2	6.09[5.77,6.40]	

N.A.: not available, N.S.: no significant between the two group, WMD: weighted mean difference, 95%CI: 95% confidential interval.

M stands for manner, M1:prevention manner, M2:treatment manner; U stands for usage, U1: continuous therapy of BPs, U 2: intermittent therapy of BPs; G stands for generation, G1: the first generation of BPs, G2: the second and third generation of BPs; Ca stands for calcium, Ca1: supplemented with calcium, Ca2: not supplemented with calcium; RA: only RA patients were included, RD: patients with all sort of rheumatic diseases were included; in mean age, Age1 : more than 50 years, Age2: less than 50 years; in Jadad score, High: ≥3 points, Low: <3 points; B stands for blinding, B1: blinding to patients; B2: not blinding to patients.

Sensitive analyses were performed based on methodological quality (Jadad score and blinding). The RR reduction of vertebral fracture was comparable between low quality trials and high quality trials, so did the WMD of lumbar spine BMD percent change (Table 2). Similarly, there was no statistical difference of effect size between blinding and non-blinding subgroup (Table 2).

### Adverse Reactions

Gastrointestinal symptoms, such as dyspepsia, vomiting and nausea, were common adverse events reported in trials. The incidence of adverse events was not different between BPs group and control group (Figure S2a). There were more withdrawals due to side effects in BPs group relative to control group (P = 0.02) (Figure S2b).

## Discussion

The current meta-analysis is a first to assess the efficacy of BPs in the prevention of vertebral and non-vertebral fractures in patients with rheumatic diseases. Evidence supports that BPs reduce the risk of vertebral fractures in rheumatic patients. This benefit can be observed at 18 months when using BPs for osteoporosis prevention and at 36 months for osteoporosis treatment. Rheumatic patients using BPs tended to have a lower incidence of non-vertebral fracture than those not taking BPs, though this tendency did not reach statistical significance in the current study. Moreover, BPs prevent bone loss at lumbar spine, hip, and femoral neck and this efficacy increases over time during the middle-term follow-up. As for managing osteoporosis in rheumatic patients, using BPs for prevention purpose has more benefits than for treatment purpose.

Although the consequences of osteoporosis, such as pain, fractures, disability and even death, are well known, using BPs to prevent and treat such complications in rheumatic patients is uncommon [50], [51]. Even the supplement of calcium and vitamin D was administered in less than one third patients requiring GC therapy [52]. Typically, calcium and vitamin D are often the only remedy for osteoporosis in rheumatic patients, if any. There is increasing evidence supports that calcium and vitamin D are insufficient to prevent bone loss in rheumatic patients who are treated with high dosage of GC [34], [41], [42], [44], [45], [53]. Although vitamin D and calcitonin can inhibit GC-induced bone loss in lumbar spine [54], [55], they are inferior to BPs, as concluded in two other meta-analyses [55], [56].

By analyzing previous studies of relatively high quality, this comprehensive meta-analysis also revealed that rheumatic patients benefit more from BPs than from calcium, vitamin D, or calcitonin in preventing and treating osteoporosis. We thus suggest that BPs had better be used routinely in rheumatic patients, especially who start GC therapy with a high dosage, while calcium and/or vitamin D as adjuncts could also be considered.

There are some advantages in our study. To minimize the confounding from the original disorders, we include only rheumatic patients, with inclusion and exclusion criteria rigidly defined before literature search. Twenty RCTs with 1422 patients from more than 10 countries were included for quantitative analysis, and intent-to-analysis data were used in mostly trials. Additionally, results presented in the current study were not significantly changed by further excluding relatively low quality trials and non-blinding trials. Conclusions drew, therefore, may represent the best of currently available trials and may be generalized in clinical practice.

BPs are important to prevent osteoporotic fractures in post-menopausal women [57]. In this study, we also found that BPs can reduce the risk of vertebral fractures in rheumatic patients. Moreover, we noticed that the effect of BPs occurred at 18 months when using BPs for osteoporosis prevention, and 36 months when using BPs for osteoporosis treatment. One interpretation is that fractures always occurred sometime later after osteoporosis is established. Clinical trials of one or two years follow-up may not be adequately powerful to detect a statistical difference. Another explanation is that patients who started GC therapy at high dosage tended to predispose to fractures [6], [7]. Interestingly, we also found that the effect size of BPs in lumbar spine BMD change considerably increased from 3.64% at 24 months to 5.87% at 36 months. This supports our view that a significant effect of BPs in preventing vertebral fractures may occurred at 36 month. Furthermore, we noted that multiple vertebral fractures occurred more frequently in patients treated with placebo than those with BPs [19], [34], [42].

In patients treated with BPs, non-vertebral fractures only involved small bones of the extremities, such as the wrist and phalangal bones. On the other hand, hip and tibia fractures with serious consequences, were reported in patients without BPs treatment [22], [40]. This can be attributed to BPs, as risedronate can significantly reduce the risk of hip fractures among osteoporotic women [58], [59]. Anyway, Long-term trials are required to evaluate this effect of BPs in the prevention of non-vertebral fractures.

A previous meta-analysis, studying trials of 12 months follow-up, revealed that BPs can maintain lumbar spine BMD with a WMD of 4.3% for patients requiring GC treatment [17]. In our study, however, the effect size was 3.29% at 12 months. This may be due to the difference of subjects studied, while the participants in previous study suffered from a variety of diseases requiring GC, only patients with rheumatic diseases were included in the current study. A lot of factors related to rheumatic diseases, such as disease activity, inflammatory factors, immobility may also induce bone loss [8]–[14] and therefore, responsible for the difference between the WMDs observed. Furthermore, by including RCTs with middle-term follow-up, we were able to evaluate the middle-term efficacy of BPs, and observed that the WMD increased gradually over time.

An interesting finding is that the efficacy of BPs therapy was much greater in prevention trials than treatment trials. The reason maybe that patients treated with GC typically undergo a rapid bone loss within the first 3 months and reach a peak at 6 months, then enter into a slower and steady bone loss process [50], [51]. The finding highlights the importance of using BPs for osteoporosis prevention rather than for osteoporosis treatment in patients with rheumatic diseases.

In our study, The first generation of BPs tended to have better effects in reducing the risk of vertebral fractures at 6 and 12 months than the second and third generation of BPs (Table 2). This tendency, however, was not observed at 24 and 36 months when only treatment trials were analyzed. The efficacy of administered way of BPs, continuous or intermittent, remains controversial in literatures. While Chesnus and Luckey et al reported that continuous or intermittent therapy of some BPs made no difference in treating postmenopausal osteoporosis [60], [61], some other authors suggested that the continuous therapy was superior to intermittent therapy [20], [36]. From our subgroup analysis we observed that there was no superiority of continuous therapy over intermittent therapy. As intermittent therapy is more affordable and may have less adverse events than continuous therapy, this topic deserves further investigation.

Surprisingly, BPs was less effective in decreasing risk of vertebral fractures for RA patients than for patients with other rheumatic diseases, but the efficacy on improving lumbar spine BMD was comparable. Therefore, this result should be treated with cautions, and it is not adequately powerful to make disease-specific recommendations. Calcium and/or vitamin D are common concomitant drugs for BPs therapy. In current subgroups analyses, we did not observe benefit of calcium and/or vitamin D as supplementation.

There are some limitations in our study. First, as all the included trials were RCTs, the sample sizes of mostly trials were relatively small. This may underestimate the effect of BPs, particularly in subgroup analyses. Also, we did not study the change of biochemical markers of bone turnover. Neither did we focus on the efficacy of a special BPs in treating osteoporosis in rheumatic patients. Such studies, if available, are crucial in guiding the management of osteoporosis in rheumatic patients. Finally, since there is only one trial enrolling rheumatic patients without GC treatment, the conclusion may relatively restricted that it is applicable for rheumatic patients who did receive glucocorticoid treatment.

## Conclusions

In both short- and middle-term therapy, BPs are effective agents in preserving bone mass from loss for patients with rheumatic diseases, mainly for those who have GC consumption. BPs can prevent bone loss at both lumbar spine and hip, and can further reduce the risk of vertebral fractures. Moreover, the efficacy of BPs is better when using BPs to prevent rather than to treat osteoporosis in rheumatic patients. There is, however, no robust evidence to suggest that BPs can prevent non-vertebral fractures and that continuous therapy is better than intermittent therapy. RCTs of large sample size trials with long-term follow-up are needed to further determine the efficacy and optimal usage of BPs in managing osteoporosis in patients with rheumatic diseases.

## Supporting Information

Figure S1 **A funnel plot of the trials with vertebral fractures as the outcome.**
(TIF)

Figure S2 **Pooled estimate for the relative risk of gastrointestinal adverse events (a) and withdrawals (b).**
(TIF)

Table S1 **Characteristics of included trials.**
(DOC)

Checklist S1 (DOC)
