# Supplementary figures and images for: Bisphosphonates for the Prevention and Treatment of Osteoporosis in Patients with Rheumatic Diseases: A Systematic Review and Meta-Analysis
Source: PLoS One. 2013 Dec 6;8(12):e80890. doi: 10.1371/journal.pone.0080890 (PMC3855695; doi:10.1371/journal.pone.0080890)

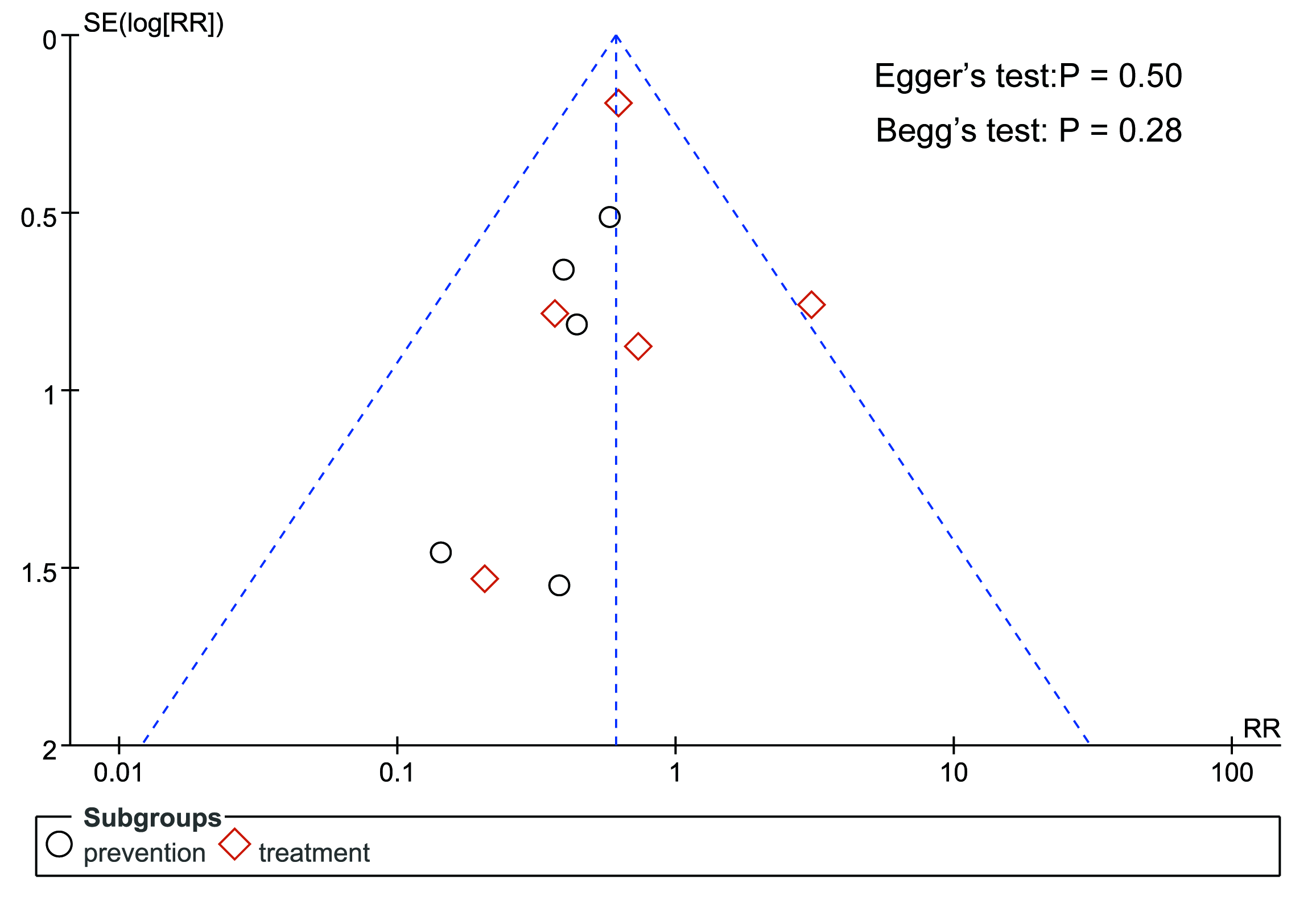

Supplement: Figure S1 — A funnel plot of the trials with vertebral fractures as the outcome. (TIF) [file pone.0080890.s001.tif]

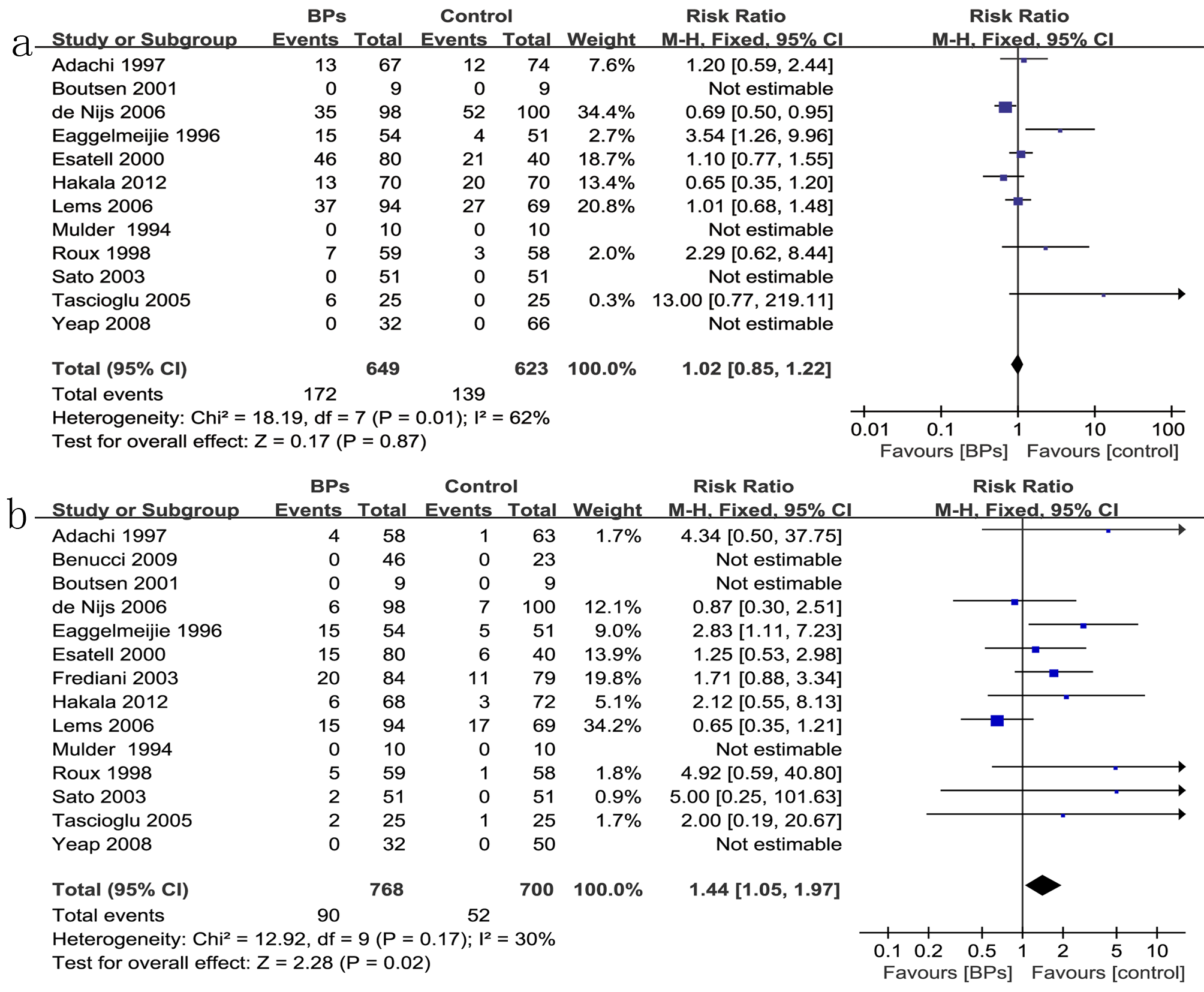

Supplement: Figure S2 — Pooled estimate for the relative risk of gastrointestinal adverse events (a) and withdrawals (b). (TIF) [file pone.0080890.s002.tif]
