# Supplementary material for: Bisphosphonates for the Prevention and Treatment of Osteoporosis in Patients with Rheumatic Diseases: A Systematic Review and Meta-Analysis
Source: PLoS One. 2013 Dec 6;8(12):e80890. doi: 10.1371/journal.pone.0080890 (PMC3855695; doi:10.1371/journal.pone.0080890)
Supplement: Table S1 — Characteristics of included trials. (DOC) [file pone.0080890.s003.doc]

**Table S1: Characteristics of included trials**

| **Source** | **No. of patients/**  **follow-up(months)** | **Mean age**  **(years)** | **Inclusion criteria** | **Intervention** | **Generation of BPs** | **Calcium**  **supplement** | **Jadad score** | **Double Blinding** |
| --- | --- | --- | --- | --- | --- | --- | --- | --- |
| **Mulder**  **1994** | 20/12 | 73 | Rheumatic diseases, Prevention 1. | 1) oral cyclical etidronate 400 mg  2) only prednisolone | G1 | No | 2 | No |
| **Eaggelmeijie 1996** | 105/36 | 50.1 | RA, without GC therapy | 1) oral pamidronate 300 mg/d  2) oral placebo 150 mg/d | G2-3 | No | 5 | Yes |
| **Adachi**  **1997** | 141/12 | 61.7 | Rheumatic diseases,  Prevention 2. | 1) oral cyclical etidronate 400 mg  2) oral cyclical placebo 400 mg | G1 | Yes, 0.5 g/d | 4 | Yes |
| **Roux**  **1998** | 117/12 | 58.7 | Rheumatic diseases, Prevention 2. | 1) oral cyclical etidronate 400 mg  2) oral cyclical placebo 400 mg | G1 | Yes, 0.5 g/d | 4 | Yes |
| **Jenkins**  **1999** | 28/13 | 67.4 | RA , PMR,  Prevention 1. | 1) oral cyclical etidronate 400 mg  2) oral cyclical placebo | G1 | Yes, 0.5 g/d | 4 | Yes |
| **Eastell**  **2000** | 120/36 | 63.6 | RA,  Treatment 1 | 1) daily risedronate 2.5 mg/d  2) cyclical risedronate 15 mg/w  3) daily placebo | G2 | No | 3 | Yes |
| **Boutsen**  **2001** | 27/12 | 57.0 | Rheumatic diseases, Prevention 1. | 1) i.v. 90 mg pamidronate  2) i.v. 90 mg pamidronate first time, then 30 mg once every 3 months  3) calcium 0.8g/d | G2 | Yes, 0.8 g/d | 3 | No |
| **Sato**  **2003** | 102/36 | 43.4 | Rheumatic diseases, Treatment 2 | 1) oral cyclical etidronate 200 mg 2) alfacalcitridol 0.75 µg/d and calcium 3.0 g/d for every 90 day | G1 | Yes, 3.0 g/d, alfacalcidol 0.75 µg/d | 3 | No |
| **Frediani**  **2003** | 163/48 | 61.7 | RA and PsA,  Treatment 2 | 1) i.m. clodronate100 mg/week, plus calcium and vitamin D  2) calcium 1 g/d and vitamin D 800 UI/d | G1 | Yes,1g/d,  vitamin D 800 UI/d | 2 | No |
| **Nakayamada 2004** | 21/12 | <50 | Rheumatic diseases, Prevention 1 | 1) oral cyclical etidronate 200 mg  2) oral cyclical placebo 200 mg | G1 | No | 1 | No |
| **Tascioglu**  **2005** | 50/24 | 57.0 | RA,  Treatment 2 | 1) oral alendronate 10mg/d  2) calcitonin 200IU/d | G2 | Yes, 1g/d, vitamin D 400 IU/d | 2 | No |
| **Lems**  **2006** | 163/12 | 61.7 | RA,  Treatment 1 | 1) oral alendronate 10mg/d  2) placebo 1 μg/d | G2 | Yes, 0.5 or 1 g/d, vitamin D 400 UI/d | 4 | Yes |
| **de** **Nijs**  **2006** | 201/18 | 60.1 | Rheumatic diseases,  Prevention 2 | 1) oral alendronate 10mg/d and placebo  2) alfacalcidol 1μg/d and placebo | G2 | Yes, 0.5 g/d | 5 | Yes |
| **Yamada**  **2007** | 12/12 | 70.6 | RA,  Treatment 1 | 1) oral risedronate 2.5 mg/d  2) alfacalcidol0.5 μg/d | G2 | Yes | 2 | No |
| **Yeap**  **2008** | 98/24 | 30.1 | SLE,  Treatment 2 | 1) oral alendronate 70mg/week  2)calcitriol 0.25 μg twice/d  3) calcium 0.5g twice/d | G2 | Yes, 1 g/d calcitriol, 0.5μg/d | 3 | No |
| **Takeda**  **2008** | 22/24 | 47.2 | Rheumatic diseases, Treatment 2 | 1) oral alendronate 5 mg/d plus alfacalcidol 1μg/d  2) alfacalcidol 1μg/d | G2 | No | 1 | No |
| **Okada**  **2008** | 47/18 | 32.0 | Rheumatic diseases, Prevention 1. | 1) oral alendronate 5 mg/d plus alfacalcidol  2) alfacalcidol 1 mg/d | G2 | Yes, 0.6 g/d | 4 | Yes |
| **Sato**  **2008** | 91/168 | 43.7 | Rheumatic diseases, Treatment 2 | 1) oral cyclical etidronate 200mg  2) alfacalcitridol 0.75 µg/d and calcium 3.0 g/d for every 90 days | G1 | Yes, 3.0 g/d, alfacalcidol 0.75 µg/d | 3 | No |
| **Benucci**  **2009** | 69/12 | 66.9 | Rheumatic diseases,  Treatment 1 | 1) i.m. neridronate 25mg/month  2) calcium 1g/d and vitamin D 800 UI/d | G2 | Yes, 1g/d, vitamin D 800 UI/d | 2 | No |
| **Hakala**  **2012** | 140/12 | 63.5 | Rheumatic diseases,  Treatment 1 | 1) oral ibandronate 150 mg/month  2) oral placebo 150 mg/month | G2 | Yes, 1g/d, vitamin D 400 UI/d | 4 | Yes |

G1: the first generation of BPs, G2-3: the second and third generation of BPs, d: day, i.v.: intravenous injection, i.m.: intramuscular injection.

Prevention 1: starting BPs treatment at the initiation of GC therapy.

Prevention 2: starting BPs treatment at initiation of GC therapy or within 3 months GC therapy.

Treatment 1: BPs treatment for long-term GC user, mean dosage < 7.5mg/d.

Treatment 2: BPs treatment for long-term GC user, mean dosage>7.5mg/d.

Oral cyclical etidronate 200mg or 400mg: oral cyclical etidronate 200mg or 400 mg daily for 2weeks, followed by control (placebo, calcium and/or vitamin D) for 11weeks.
